# Aggregate complexes of HIV-1 induced by multimeric antibodies

**DOI:** 10.1186/s12977-014-0078-8

**Published:** 2014-10-02

**Authors:** Daniel J Stieh, Deborah F King, Katja Klein, Pinghuang Liu, Xiaoying Shen, Kwan Ki Hwang, Guido Ferrari, David C Montefiori, Barton Haynes, Punnee Pitisuttithum, Jaranit Kaewkungwal, Sorachai Nitayaphan, Supachai Rerks-Ngarm, Nelson L Michael, Merlin L Robb, Jerome H Kim, Thomas N Denny, Georgia D Tomaras, Robin J Shattock

**Affiliations:** Center for Infection, Department of Cellular and Molecular Medicine, St George’s, University of London, London, SW17 0RE UK; Current address: Department of Cellular and Molecular Biology, Northwestern University, Feinberg School of Medicine, Chicago, IL 60611 USA; Mucosal Infection & Immunity Group, Section of Infectious Diseases, Imperial College London, St Mary’s Campus, London, W2 1PG UK; Duke Human Vaccine Center, Duke University Medical Center, Durham, NC 27710 USA; Faculty of Tropical Medicine, Mahidol, Thailand; Armed Forces Research Institute of Medical Sciences, Bangkok, Thailand; Ministry of Public Health, Bangkok, Thailand; Military HIV Research Program, Walter Reed Army Institute of Research, Silver Spring, Maryland, United States of America; Current address: Division of Swine Infectious Diseases, Harbin Veterinary Research Institute, Chinese Academy of Agricultural Sciences, Harbin, 150001 China

**Keywords:** HIV-1, Mucosal immunity, Immunoglobulin A, Aggregation

## Abstract

**Background:**

Antibody mediated viral aggregation may impede viral transfer across mucosal surfaces by hindering viral movement in mucus, preventing transcytosis, or reducing inter-cellular penetration of epithelia thereby limiting access to susceptible mucosal CD4 T cells and dendritic cells. These functions may work together to provide effective immune exclusion of virus from mucosal tissue; however little is known about the antibody characteristics required to induce HIV aggregation. Such knowledge may be critical to the design of successful immunization strategies to facilitate viral immune exclusion at the mucosal portals of entry.

**Results:**

The potential of neutralizing and non-neutralizing IgG and IgA monoclonals (mAbs) to induce HIV-1 aggregation was assessed by Dynamic light scattering (DLS). Although neutralizing and non-neutralizing IgG mAbs and polyclonal HIV-Ig efficiently aggregated soluble Env trimers, they were not capable of forming viral aggregates. In contrast, dimeric (but not monomeric) IgA mAbs induced stable viral aggregate populations that could be separated from uncomplexed virions. Epitope specificity influenced both the degree of aggregation and formation of higher order complexes by dIgA. IgA purified from serum of uninfected RV144 vaccine trial responders were able to efficiently opsonize viral particles in the absence of significant aggregation, reflective of monomeric IgA.

**Conclusions:**

These results collectively demonstrate that dIgA is capable of forming stable viral aggregates providing a plausible basis for testing the effectiveness of aggregation as a potential protection mechanism at the mucosal portals of viral entry.

**Electronic supplementary material:**

The online version of this article (doi:10.1186/s12977-014-0078-8) contains supplementary material, which is available to authorized users.

## Background

The induction of protective antibodies at mucosal portals of virus entry may be essential for vaccine efficacy against HIV-1 acquisition. Ideally, vaccination would induce broadly neutralizing antibodies (bnAb) in the fluids present at susceptible mucosal surfaces such as the lower female genital tract and the rectum [1,2]. However this type of antibody response has yet to be generated by current vaccine candidates and will likely require new immunogens and vaccination strategies yet to be realized. Furthermore, it remains unclear whether classical neutralization is a prerequisite for mucosal protection or if other antibody functions can reduce the risk of mucosal HIV-1 infection.

This critical question has been thrown in sharp relief following the immune-correlates analysis of the human RV-144 Thai efficacy trial that demonstrated a modest reduction in risk of HIV acquisition (31.2%) in the absence of an efficient neutralizing antibody response. Here reduced acquisition of HIV infection correlated, not with neutralizing activity, but with non-neutralizing binding antibodies to variable regions 1 and 2 (V1V2) of the HIV-1 envelope proteins (Env). Whether such responses are a surrogate or mechanistic correlate of protection has yet to be defined. Surprisingly, high systemic levels of specific IgA, predominantly to the C1 region of Env, may have mitigated the effects of protective antibodies most likely by perturbing antibody dependent cellular cytotoxicity (ADCC). While systemic responses in the RV144 trial have been studied in detail, the extent to which they reflect mucosal responses in terms of specificity, function or isotype has not assessed and is the focus of on-going immunogenicity studies. Nevertheless these data highlight the need to explore additional antibody functions that may have protective efficacy at mucosal surfaces [3].

In addition to classical neutralization, mucosal antibodies may impede viral transfer across mucosal surfaces by inducing viral aggregation, hindering viral movement in mucus, preventing transcytosis, or reducing inter-cellular penetration through the epithelia, thereby limiting access to susceptible mucosal CD4 T cells and dendritic cells. These functions may work together to provide effective immune exclusion of virus from mucosal tissue [4,5], and would supplement Fc-receptor mediated phagocytic uptake and degradation of opsonized virus, together with potential ADCC mediated elimination of any initial foci of infection. However, these functional activities are critically modulated by immunoglobulin isoforms that differ in proportion and in structure to those in blood, and by specificity, which while showing considerable overlap to systemic antibody, may also recognize distinct epitopes [6].

Typically IgA has been associated with immune exclusion at mucosal surfaces. In the circulation, the two isoforms of IgA (IgA_1_ and IgA_2_) are predominantly expressed in monomeric form. However at mucosal surfaces both isoforms are expressed as dimer or polymers of monomeric IgA linked by an integral joining chain (J chain) [7]. Binding of the J chain to the polymeric Ig receptor (pIgR) triggers active transport across mucosal epithelial cells and luminal release following cleavage of pIgR, the mature secretory IgA (scIgA) still complexed to the cleaved portion of pIgR termed the secretory component (SC) [8]. In contrast, both systemic and mucosal IgG isoforms (IgG_1-4_) are exclusively monomeric. Mucosal transfer of IgG occurs by passive diffusion and/or active transport mediated by the neonatal Fc receptor (FcRn). The relative proportions of IgG and scIgA isotypes differ by mucosal compartment where IgG predominates in vaginal fluids, while IgA dominates in rectal secretions [9,10].

Although aggregation of HIV particles has been proposed as a potential mechanism for decreased incidence of initial infection [11-13], this has not been tested experimentally. Furthermore the antibody characteristics required for effective HIV aggregation have not been defined, where the ability to form even quasi-stable higher order complexes might be a relevant factor in conferring protection by decreasing the number of infectious units per inoculum. Indeed, other mucosal viruses including poliovirus, rhinovirus, and picornavirus are known to be susceptible to neutralization by aggregation [14-17]. To the best of our current understanding, these types of complexes have not been documented with HIV.

To determine the existence and feasibility of generating viral aggregates by antibody cross-linking we have tested the potential of neutralizing and non-neutralizing IgG and IgA monoclonals from infected subjects as assessed by Dynamic Light Scattering (DLS). This technique uses the degree of Brownian motion in a liquid system to determine the size of particles and their heterogeneity by measuring over time intervals the interference pattern of photons deflected from a laser interacting with suspended particles in solution, reviewed by Hassan et al. [18]. This is known as a time-correlation function. Large particles have a stable correlation over longer time frames. Small particles move more rapidly, resulting in an interference pattern more dissimilar over a smaller time interval.

Env-specific antibodies of the IgG class were found to be incapable of forming viral aggregates, although able to cross-link recombinant trimeric Env protein. However polymeric IgA monoclonals were able to efficiently aggregate HIV-1 into discreet complexes. Furthermore, assessment of post-vaccination IgA serum samples from the RV144 Thai vaccine trial demonstrated significantly larger particle formation than pre-vaccination IgA. Knowledge of the antibody isotype and specificity needed to induce aggregation may allow more specifically tailored design of immunization strategies to facilitate immune exclusion at the mucosal portals of entry.

## Results

### HIV-1 Env specific IgG mAbs aggregate trimeric Env protein but fail to induce viral aggregation

Initial experiments were performed to assess the ability of HIV-1 Env specific monoclonal IgG antibodies to aggregate recombinant HIV-1 Env protein providing a classical two species system (antigen-antibody). A panel of Env-specific monoclonal antibodies (mAbs) was assessed for the ability to aggregate soluble trimeric gp140 (CN54 gp140) using dynamic light scattering (DLS) to determine particle size. gp140 better represents the envelope spike of HIV than monomeric gp120 and its trimeric structure displays three potential binding sites per molecule. This protein was chosen because if its ready availability, stability profile and our previous experience studying it’s biochemical properties [19,20]. The rate of change in the time-correlation function was used to calculate the heterogeneity within a sample, known as the polydispersity index or PDI. A sample size ranges from 0 for a uniform particle size to 1 for a random dispersion. This alone is not a measure of aggregation, but gives insight into changes in the composition of a sample, which tends to correspond with formation of higher order complexes. mAbs alone ranged in size from 4-12 nm, while soluble gp140 measured 12.4 ± 2.4 nm, with a PDI of 0.17.

Many of the antibodies specific for the outer domains of Env were able to induce aggregation of trimeric protein (Table 1, Column “gp140 Aggregation”). To test if this effect could be saturated, the stoichiometry was varied. mAbs were added at a range of concentrations (0.5 - 50 μgml^−1^) to account for the fact that aggregate formation is optimal at specific stoichiometry, related to antibody affinity. The ratio of antibody to Env binding sites ranged from a maximum of 11.5:1 to a minimum of 1:8.67. Aggregate formation was observed with most antibodies. Optimal aggregate formation was most common near a Ab:Env ratio close to 1. Eighteen of the mAbs tested could form aggregates at the maximal concentration of 50 μgml^−1^; at 0.5 μgml^−1^ aggregation was only seen with 3H12. All neutralizing antibodies (nAbs) targeting epitopes present on CN54 gp140 formed aggregates. The multiple specificities present in polyclonal HIV-Ig (pooled purified IgG from infected individuals) allow for multiple cross-linking at a wide range of concentrations and had a higher PDI. Here, the potent ability to form aggregates likely reflects the polyclonality of antibodies.

**Table 1:** **Aggregation assessment of trimeric Env or HIV-1**_**BaL**_ **virions incubated with antibody**

**Assessment of IgG antibodies for aggregation of gp140 and HIV-1**										
**Antibody**	**Epitope (residues)**	**Trimeric CN54 gp140**					**Neutralization**	**Purified HIV-1 BaL**		
		**gp140 Agg.** ^**1**^	**Conc. range** ^**2**^	**Ab Conc.**	**Ab:Env ratio**	**PDI** ^**3**^	**IC**_**50**_	**Virus Agg.** ^**1**^	**Δ Virus size** ^**4**^ **(nm)**	**PDI** ^**3**^
HIV-Ig	Polyclonal	++	50-1	50	17:1	0.67	10.6	**-**	19.3	0.41
1B7	gp41 (ND)	++	50-10	10	3.4:1	0.56	>50	**-**	5.4	0.23
5F3	gp41 cluster V	++	50-2	5	1.7:1	0.53	>50	**-**	12.5	0.30
24G3	gp41 (526-543)	++	50-5	5	1.7:1	0.49	>50	**-**	10.6	0.26
3H12	gp41 (ND^5^)	++	50-.5	2	1:1.5	0.45	>50	**-**	4.0	0.33
b12	gp120 CD4bs	++	50-2	5	1.7:1	0.54	0.75	**-**	12.7	0.34
G8	gp120 (dis)	++	5-2	5	1.7:1	0.51	>50	**-**	−6.4	0.21
1F7	gp120 V3	++	50-1	5	1.7:1	0.48	1.24	**-**	6.3	0.32
25C2	gp41 CD4i (526-543)	+	50-5	10	3.4:1	0.35	>50	**-**	−1.6	0.34
1H5	gp41 (579-613)	+	50-2	2	1:1.5	0.32	>50	**-**	1.0	0.23
4B3	gp41 (579-613)	+	50-5	5	1.7:1	0.31	>50	**-**	−4.3	0.28
1B1	gp120 (CD4bs dis)	+	50-10	10	3.4:1	0.37	>50	**-**	7.4	0.27
3D5	gp120 (ND)	+	50-5	5	1.7:1	0.34	>50	**-**	−12.4	0.21
2G6	gp120 (CD4bs dis)	+	10-5	10	3.4:1	0.32	>50	**-**	1.4	0.26
3B7	gp120 (non-V3)	+	50-5	10	3.4:1	0.28	>50	**-**	12.6	0.31
4D4	gp41 (579-613)	±	10	10	3.4:1	0.30	>50	**-**	16.2	0.28
1F11	gp41 (579-613)	±	10	10	3.4:1	0.27	>50	**-**	2.2	0.40
3D6	gp41 (604-617)	±	50-5	5	1.7:1	0.23	>50	**-**	9.8	0.27
2G12	gp120 (C2-C3-V4 glycan)	±	50-10	10	3.4:1	0.19	1.89	**-**	13.7	0.26
7B2	gp41 cluster I	±	50	50	17:1	0.27	9.65	**-**	11.0	0.38
2F5	gp41 MPER (662-667)	-	N/A	N/A	N/A	0.21	2.73	**-**	14.6	0.37
4E10	gp41 MPER (824-830)	-	N/A	N/A	N/A	0.20	>50	**-**	−1.5	0.22

The average size of the preparations was measured by dynamic light scattering. Data is the average of three experiments, each measured in triplicate. For CN54gp140, Ab concentration, Ab:Env ratio and PDI are reported for optimal aggregate formation conditions. For HIV-1 BaL, Δ Virus size and PDI are reported for 10 μgml^−1^. All concentrations are in μgml^−1^.

^1^Aggregate formation was scored by the maximum size measured: ++ for particles > 100 nm; + for 40 ≤ 100 nm; ± for 20 < 40 nm, – for ≤ 20 nm.

^2^Concentration range where the gp140-Ab complexes scored positive for aggregation.

^3^Relative heterogeneity of preparations is evaluated by polydispersity index (PDI).

^4^Change from purified virus particle size of 150.2 nm.

^5^ND: not determined.

To correlate Env aggregation with neutralizing activity, the potency of these antibodies was tested against HIV-1_BaL_, a CCR5 tropic lab-adapted clade B strain of HIV-1, in a TZM-bl assay. These cells express HIV1-1 receptors, are highly susceptible to infection, and produce luciferase in response to infection. Antibody concentrations were used at the same levels as those capable of aggregating the Env trimer. The majority of mAbs failed to inhibit infection (Table 1, Column “IC_50_”). The ability of mAbs to induce complexes amongst Env trimers was not predictive of the ability to block viral infection.

To test the ability of Env-specific antibodies to form higher order complexes with whole virions, highly purified HIV-1 was incubated with the same panel of mAbs. Purification of viral particles by centrifugation has been shown to retain functional Env in its native conformation [21-23]. Purified viral particles alone had an average diameter of 150.2 ± 2.1 nm, with a PDI of 0.15, indicating a homogeneous population of particles. The liquid phase measurement is larger than that traditionally defined by electron microscopy (~120 nm) where sample processing can condense the viral membrane [21,24].

Incubation of purified virions with the panel of Env specific IgGs had uniform results: the maximum change in size of IgG treated virions was no more than twice the size of an antibody (Table 1, Column “Virus Aggregation”), with a modest increase in PDI. These results reflect the ability of antibodies to bind and opsonize virions, but indicate that each arm of an IgG molecule was not able to stably bind and bridge between different virions.

Lack of viral aggregation was not due to inability of antibody to bind whole virions. To confirm that IgG treated virions were effectively opsonized and susceptible to aggregate formation, free anti-Env mAb was removed using a 250 kD spin column, and a secondary anti-human Fc antibody was added. This resulted in appreciable aggregation with monoclonal (1F7), and polyclonal (HIV-Ig), as well as antibody specific for host derived protein (HLA-DR) (Additional file 1: Figure S1). These results indicate that steric or flexibility limitations of IgG molecules restrict aggregate formation with virions.

### In contrast to IgG, Env specific dimeric IgAs can induce aggregation of HIV-1

The inability of all IgG class antibodies tested to aggregate virions suggests they would be ineffective at mediating immune exclusion by viral aggregation. Because the dIgA structure affords increased valency, antibodies of this class were next investigated. 2F5 was available as monomeric and dimeric IgA_1_ and b12 as IgA_2_ mAbs. Also available were an additional panel of 3 IgA_1_ isolated from patients’ serum from the CHAVI 001 cohort: ACL4, E10, and XA1 which contain mainly mIgA.

The well-characterized 2F5 mAbs binds to the MPER of gp41 as well as interacting with the lipid component of the viral membrane, while b12 binds to CD4 binding site in gp120. To ensure that class switching of these antibodies did not alter their affinity for their epitope, kinetic binding measurements between purified virions and mAbs of the IgG, IgA, and IgM class were made. The resulting affinities varied between CCR5- (Additional file 2: Figure S2A) and CXCR4-tropic (Additional file 2: Figure S2B) viral strains although class switching antibodies altered the binding only modestly. Affinities ranged from 10.8nM for b12 IgG binding HIV-1_BaL_ to 104nM for 2F5 IgM binding HIV-1_RF_.

In sharp contrast to treatment with Env specific IgG, DLS measurements on virions treated with 10 ugml^−1^ 2F5 dIgA_1_ showed significant levels of viral aggregation (Table 2). The average diameter of the preparation increased from 150 nm to 225 nm and resulted in a four fold increase in PDI from 0.15 to 0.63. These measurements combined indicate the presence of higher order complexes. 2F5 pIgM provided a less extensive increase in particle size of 32.1 nm and a PDI of 0.41, indicating that increasing mAb valency further was not sufficient to induce more potent aggregation. Measurements made with b12 dIgA_2_ antibody resulted in similar increases in size with an increase diameter from 150 nm to 213 nm and an increased PDI to 0.67. Screening the remaining monomeric IgA antibodies failed to show significant aggregate formation.

**Table 2:** **Env-specific antibodies of the IgA class were incubated purified HIV-1 and particle size determined by dynamic light scattering**

**Assessment of aggregation of HIV-1**_**BaL**_ **by IgA-class mAbs specific for Env**				
**Antibody**	**Isotype**	**Virus Aggregation** ^**1**^	**Δ Viral Size (nm)**	**PDI**
2F5	IgG	-	14.6	0.19
	dIgA	++	75	0.63
	pIgM	+	32.1	0.41
b12	IgG	-	12.7	0.27
	dIgA	++	63	0.67
ACL4	mIgA	+	26.9	0.29
E10	mIgA	-	17.3	0.36
XA1	mIgA	-	−4.1	0.21

The change in the size of viral particles from virus alone (150.2 nm), and the polydispersity index (PDI) of virus-Ab preparations assesses the presence of aggregates.

^1^Aggregate formation was scored by the maximum size measured: ++ for ∆ > 50 nm; + for 25 ≤ 50 nm; – for ≤ 25 nm.

### Separation of viral aggregates allows specific characterization of their size and interpretation of the number of virions per complex

DLS techniques on their own are restricted in that they are not optimal for characterization of samples comprised of diverse size particles in the same preparation. In order to better characterize the degree and heterogeneity of viral aggregates induced by Env specific dIgA, separation of the sub-populations present in each preparation was necessary. This was achieved by size-exclusion chromatography (SEC) system followed by in-line DLS measurements. SEC can separate antibody from uncomplexed virions and different degrees of aggregation, enabling increased precision in sizing measurements.

Purified virions incubated with 2F5 IgG were seen to elute as a single species, and measure 176 ± 6 nm, with a PDI of 0.16 (Additional file 3: Figure S3). Three in-line detectors were employed; the infrared (IR), ultraviolet (UV), and DLS detector agreed with the elution timing of 23.4-23.6 minutes and the absence of any other particles at a detectable concentration outside of the void volume.

**Figure 1:**
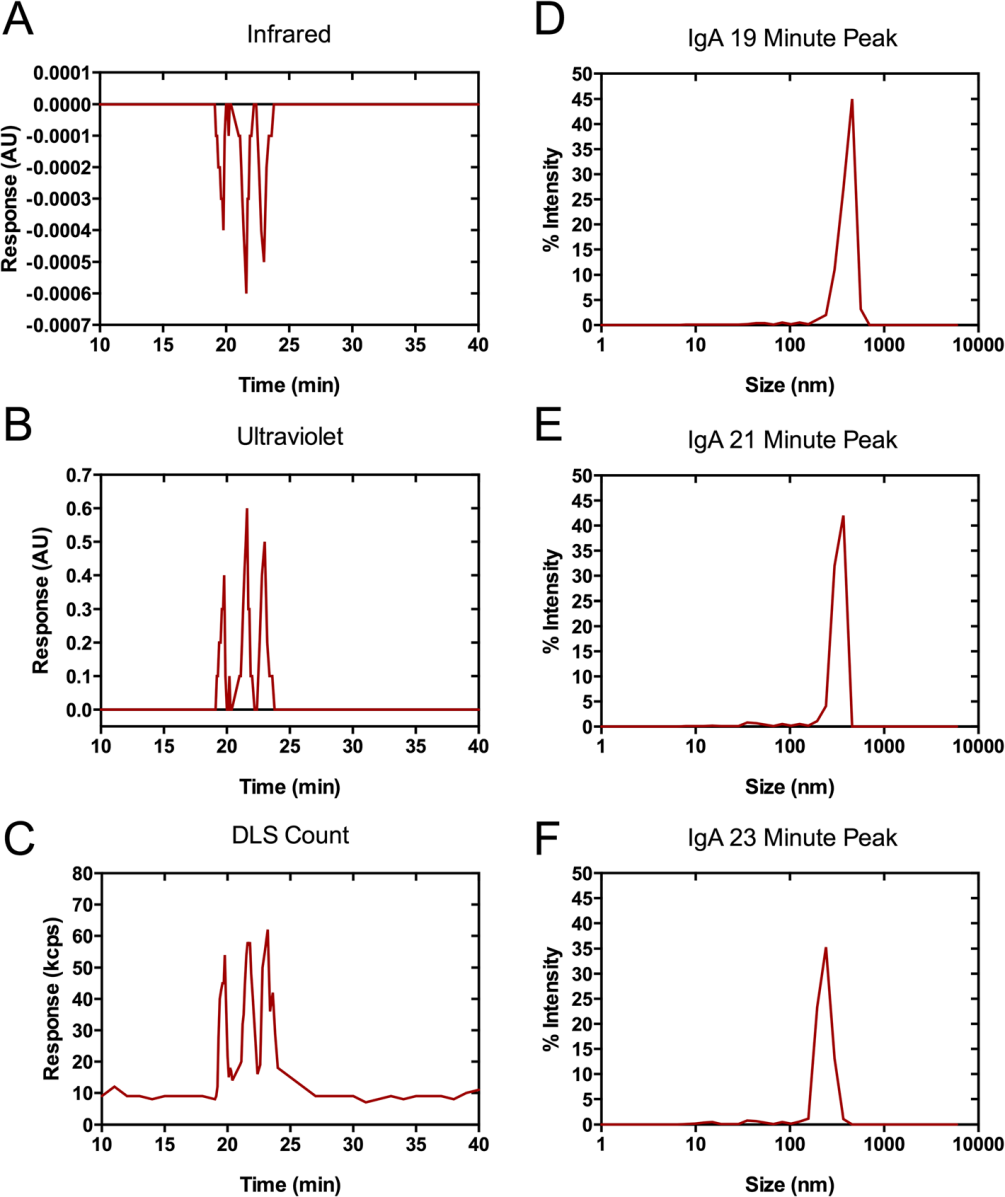
**Size-exclusion chromatography (SEC) and dynamic light scattering in tandem enable more accurate characterization of the complexes formed with 2F5 IgA.** Virus incubated with 2F5 dIgA was separated over a size exclusion column and followed by particle detection by **(A)** infrared, **(B)** ultraviolet, and **(C)** DLS detection. Size was determined for each peak eluted from the SEC system and the % of signal intensity corresponding to complex size is shown. Three peaks were detected with dIgA preparations, eluted at **(D)** 19, **(E)** 21, and **(F)** 23 minutes, measuring 450 nm, 298 nm and 180 nm, respectively. Experiments were performed in triplicate. Results shown are from one representative experiment.

In contrast, SEC-DLS of virions incubated with 2 F5 dIgA_1_ revealed three discrete populations outside of the void volume. These measured 180 ± 12 nm (PDI 0.17), 298 ± 18 nm (PDI 0.19), and 450 ± 21 nm (PDI 0.22) in diameter and eluted at 23, 21, and 19 minutes respectively (Figure 1). There was a return to baseline for each of the detectors between the particles being eluted, indicating formation of discrete complexes rather then a continuous distribution of particle sizes. Random bridging of particles would not result in separate populations being generated, but a continuous distribution. This observed ordered aggregation into discrete size fractions could provide a stoichiometric basis for understanding the mechanisms of aggregate formation.

Aggregates were then modeled using a stacked spheres methodology. Here, virions are modeled as non-overlapping spheres which fit into a volume after adjusting for the density of either close packing (ρ = 0.74) or random packing (ρ = 0.63) of virions. The number of virions in a complex (N_v_) is estimated by the volume of an aggregate (V_agg_) multiplied by the packing density (ρ) divided by the volume of a single virion *N*_*v*_ = V_agg_ × ρ × V_vir_^− 1^. This resulted in different structures being identified. The smallest complex being composed of antibody opsonized virions or 2 viral particles bound, without external antibody. The 298 nm complex is representative of 6 to 8 virions held in complex. The largest peak, with a 450 nm diameter, is indicative of between 24 and 30 virions in large complexes. The shape of complexes cannot be measured using DLS, and calculations are based on the observation that the complexes do not randomly associate, but instead form populations. The detectors returning to baseline signal between each species justified this reasoning.

The relative concentration of each species can also be estimated, based on the signal intensity corrected for the relationship between signal intensity and particle size. Larger complexes scatter a disproportionate amount of light relative to an equal number of smaller particles. Virion-2F5 dIgA preparations indicated that approximately 85% of signal was attributable to the 176 nm peak, 10% within the 298 nm peak and 5% from the largest aggregates. Normalizing for the number of virions contained within each group, 2F5 aggregation is approximately 50% efficient with 50% of virions in the 176 nm fraction, 40% in the 298 nm and 10% in the 450 nm peak.

**Figure 2:**
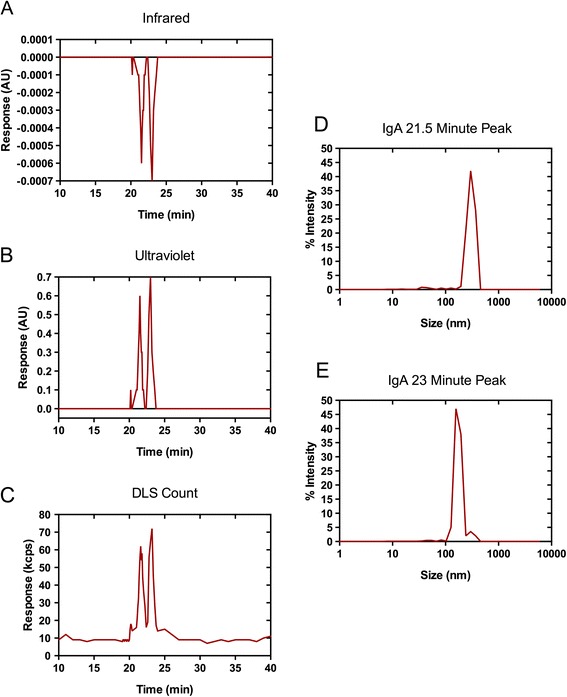
**Size-exclusion chromatography and dynamic light scattering in tandem allow more accurate characterization of viral complexes formed with b12 IgA.** Virus incubated with b12 dIgA was separated over a size exclusion column and followed by particle detection by **(A)** infrared, **(B)** ultraviolet, and **(C)** DLS detection. Size was determined for each peak eluted from the SEC system and the % of signal intensity corresponding to complex size is shown. Two peaks were detected with dIgA preparations, eluted at **(D)** 21.5 and **(E)** 23 minutes, measuring 310 nm and 180 nm, respectively. Experiments were performed in triplicate. Results shown are from one representative experiment.

Assessment of virions incubated with b12 IgG using the SEC-DLS system was similar to that with 2F5 IgG (Additional file 4: Figure S4). Virions incubated with b12 dIgA_2_ separated into two discrete populations (Figure 2), one with a mean diameter of 180 ± 21 nm, PDI 0.24, and one at 310 ± 16 nm, PDI 0.21. For b12 dIgA_2_, unlike 2F5 dIgA_1_, there was only one higher order population formed. Employing the same stacking spheres model indicates that virus with b12 dIgA forms single opsonized virions and aggregates of 6-8 virions. Correcting the for the number of individual virions contained within each fraction, b12 dIgA preparations indicated that 87-92% of total signal came from the 180 nm peak, thus 35-40% of the viral particles were aggregated.

### Serum samples from the RV144 vaccine trial show a modest ability to aggregate vaccine-matched virions

The RV144 Thai vaccine trial represents the first clinical study to demonstrate any impact on HIV-1 acquisition providing a modest protective efficacy [25]. In this trial study volunteers were vaccinated in a prime/boost regime using recombinant canary poxvirus (ALVAC-HIV) and two gp120 proteins (AIDSVAX B and E) [26]. Early analysis suggested the observed protective efficacy did not correlate with neutralizing antibody levels, or cellular immunity, and was likely affected by non-neutralizing antibody function [27,28]. Antibody responses were capable of binding infectious virions [29]. One of a number of hypotheses currently being evaluated is that virus might have been complexed with Env-specific dIgA at mucosal surfaces leading to immune exclusion. Nevertheless, high levels of HIV specific serum IgA antibodies correlated with reduced vaccine efficacy. In the systemic compartment, coating of virus with IgA may be detrimental if competing for binding with IgGs of similar specificity blocks Fcγ-Receptor mediated antiviral activity [28]. Although mucosal samples were not collected as a part of the initial RV-144 study, serum samples were collected over the course of the vaccination regime. In the absence of mucosal samples we applied our aggregation assay to the analysis of serum IgA. Levels of serum IgA are variable from one individual to another, and the amount of dimeric IgA present in human serum can range substantially, from 2 to 30% of total Ig [30], therefore serum samples were initially screened to select those with high levels of IgA binding to the autologous vaccinating Clade A/E rgp120 protein, strain A244. All of the patients in this study remained uninfected throughout the course of the vaccine trial and remain uninfected with HIV to date to the best of our knowledge. IgA was then affinity purified from the serum taken before the first (visit 1 (v1)) and after the final vaccination (visit 8 (v8)) of 29 volunteers as previously described [27,28].

To determine whether the samples contained monomeric or dimeric IgA, native western blotting with anti-human IgA-FC was performed. Both monomeric and dimeric forms of IgA were present in the pre-immunization and post-immunization samples (Additional file 5: Figure S5). IgA samples were then assessed for HIV-1 aggregation at a range of concentrations (1, 2.5 and 10 μgml^−1^). The viral strain used (A244) was matched to the clade E gp120 protein used for vaccination. Purified HIV-1_A244_ virus grown in pooled PBMCs had an average diameter of 216.5 ± 3.2 nm, and a PDI of 0.29, which was larger and more heterogeneous than that seen with the BaL virions used in previous experiments, but this was repeatable between viral preparations (Additional file 6: Figure S6A). To control for any clustering within the purified IgA preparations, total IgA was screened in the absence of any virions. These results indicated a homogenous particle size for the IgA with measurements ranging from 6 to 12 nm (Additional file 6: Figure S6B).

When pre-immunization samples (Visit 1) were incubated with HIV-1_A244_ at 10 μgml^−1^ there was no clear evidence of aggregate formation observed in any sample with a decrease in the average diameter relative to untreated virions seen in some patients. The observed decrease is caused by high levels of free antibody in solution with a diameter that is an order of magnitude smaller than virions, decreasing the mean particle size. The average size of visit 1 samples was 218.3 ± 13 nm. As visit 1 samples represent pre-immunization sera, the antibody in these preparations will not be specific for HIV-1, but will include all IgA antibodies constitutively expressed by the individual donor.

**Figure 3:**
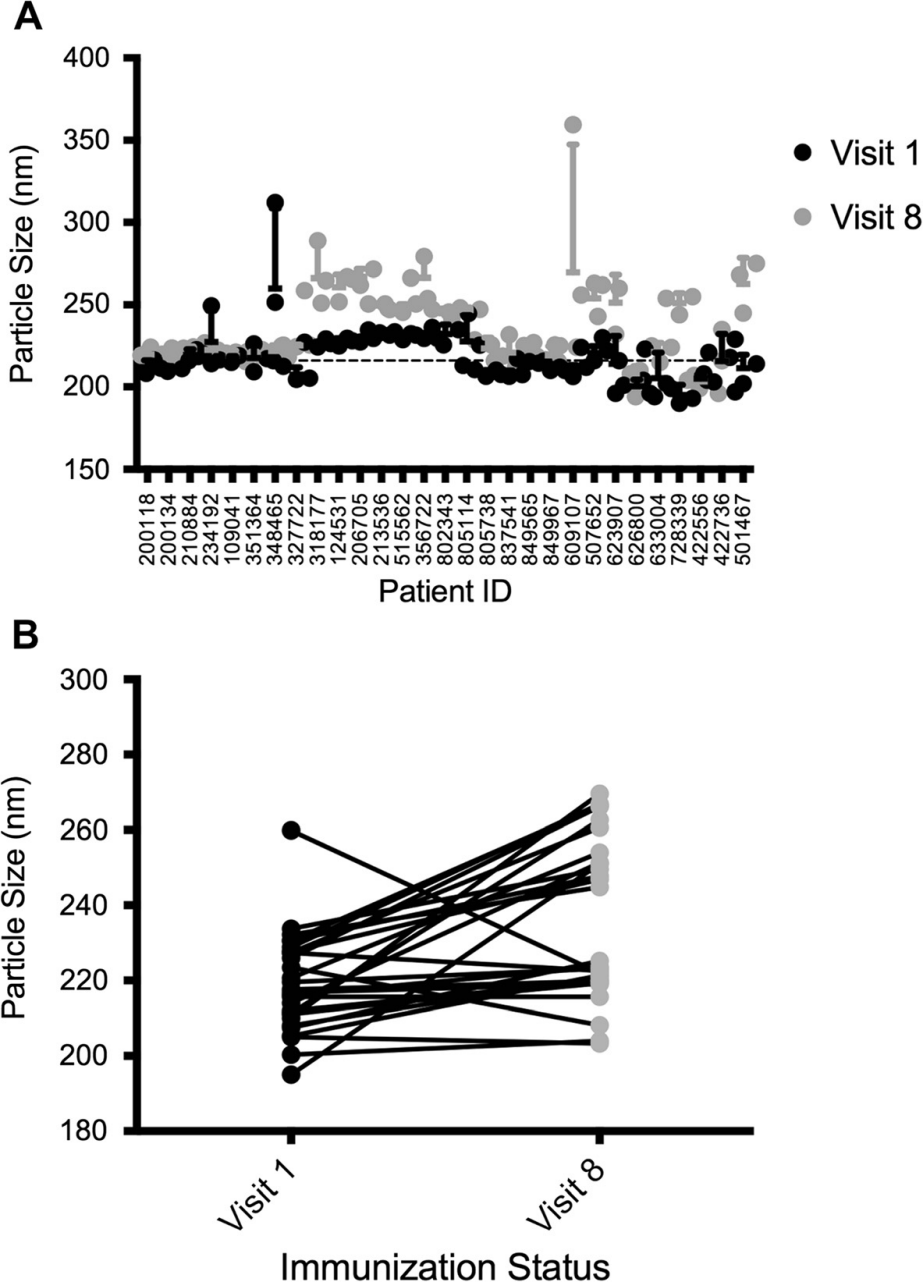
**Sizing measurements of pre-immunization compared to post-immunization samples for RV144 serum IgA. (A)** Scatter plots and **(B)** paired size measurements for pre and post immunization IgA samples incubated with HIV-1_A244_. Purified virions and IgA samples were incubated for 1 hour and measured using dynamic light scattering. Each circle shown represents the average of 3 measurements, with experiments performed three times for each sample. Horizontal dashed line indicates the size of untreated viral preparations. Error bars indicate the standard deviation within the measurements for each patient at each visit.

Final visit samples were then analyzed in the same manner. Particle size was larger with post-immunization IgA at 10 μgml^−1^ for most patients (Figure 3A). The average diameter of post-immunization samples was increased to 235.9 ± 20.5 nm. Lower antibody concentrations had no effect. The differences observed revealed a highly significant increase in the size measurements made between visit 1 and visit 8 samples (p < 0.0001) (Figure 3B) using Wilcoxon’s matched pairs test to measure significance. This indicates that there was an effect, specific to the vaccination status of this panel of individuals. When compared by repeated measures adjusted 1-way ANOVA (Bonferonni adjusted), 10 of 29 donors’ visit 8 IgAs resulted in sizes with a statistically significant increase in diameter relative to visit 1 samples (p < 0.05). One patient in particular, 728339, had a highly statistically significant (p < 0.01) increase diameter from 191 to 248 nm.

**Figure 4:**
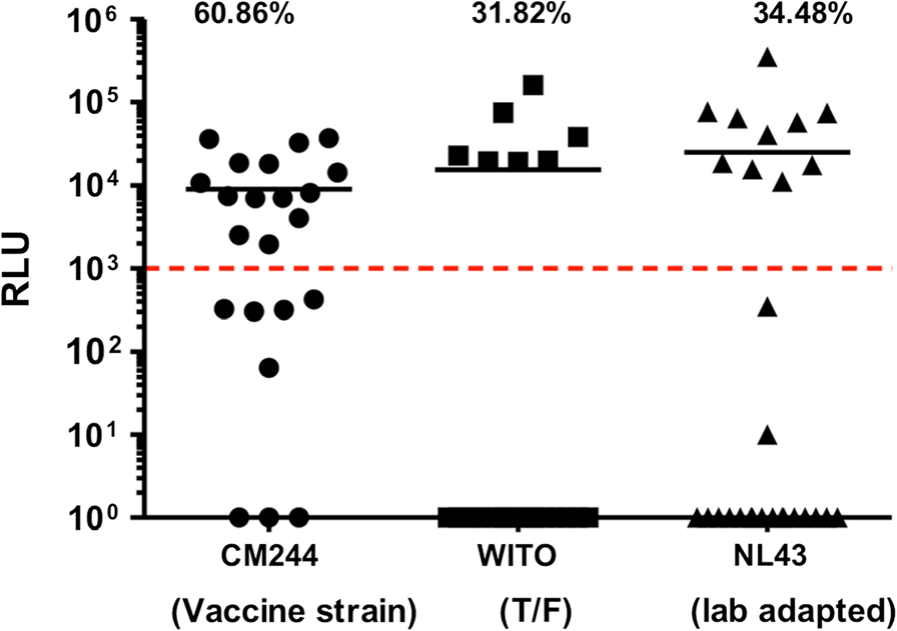
**RV144 Vaccine Induced IgA mediated Infectious HIV-1 Virion Capture.** Purified plasma IgA at visit 1 (pre-vaccination) and visit 8 (2 weeks post last vaccination) from 22 (CM244 or WITO) and 29 (NL4-3) vaccinees with detectable HIV-1 Env binding antibody responses were selected and measured in infectious HIV-1 capture assays using micro-plate based assay [29]. Relative light units (RLU) produced by infected target cells quantify the amount of infectious virus bound. Dashed line (RLU ≥1000) indicates positive samples. The percentage of tested vaccinees with virion capture capacity are 34.5% (10/29) NL4-3, and 60.9% (14/23) for lab adapted strain NL4-3 and CM244, respectively. The transmitted/founder WITO was captured by 31.8% (7/22) vaccinees. Dashed line indicates positivity threshold, defined by the background levels of capture (≥1000).

To compare changes in size with functional activity we applied an infectious capture assay to determine the ability of purified IgA to bind infectious virions. 22 (subtype A/E CM244 or transmitted/founder subtype B WITO) and 29 (lab adapted subtype B NL4-3) vaccinees with detection of HIV-1 Env binding antibody responses were selected for analysis (Figure 4). Pre-immunization samples demonstrated a background of infectious viral capture ranging from 0% to 11.1%. After immunization the percentage of tested vaccinees with infectious virion capture capacity were 31.48% (10/29) for NL4-3 and 60.86% (14/23) for vaccine strain CM244, respectively. The transmitted/founder strain WITO was captured by 31.82% (7/22) vaccinees. Pair-wise comparison (Wilcoxon matched-pair test) between pre and post-immunization IgA shows the fraction of vaccine strain subtype CM244 infectious virus capture by the antibody bound fraction increased significantly after immunization (p = 0.0003). The immune response seen amongst these participants was consistent in their ability to specifically capture infectious virus of autologous vaccinating strains of HIV-1_CM244_ [31]. However capture of infectious virions was distinct to binding to consensus soluble gp140 or gp120 from the vaccinating strain CM244 [29].

A total of 22 functional antibody assays were performed on post-vaccine serum IgG and IgA purified from the same RV144 donor pool [32]. To understand what antibody effector functions or specificities were associated with post-vaccination IgA aggregation results, a multiple regression analysis was performed against the DLS assay results (Table 3). 3 statistically significant (p < 0.05) negative correlates were identified: ConSgp140 Binding antibody multiplex assay (BAMA), capture of viral strain NL4-3 by IgA, and the A244gp120gD- BAMA assay. Pooling the antigen binding results into a “family” outcome provides a highly statistically significant negative correlate as well. There were no statistically significant positive correlations identified; the strongest predictive value was between binding of IgG to the C5 domain of gp120 (p = 0.056), although there was only enough material to perform this assay on 11 donors. No other epitope mapping, ADCC or virus neutralization results were predictive of the DLS assay. The virus capture of HIV-1_CM244_ IgA also failed to predict the DLS results, which was unexpected. The lack of association between well defined antibody functionalities and potential for aggregation indicates that the DLS assay is evaluating a unique niche in immunological activity.

**Table 3:** **Correlation between antibody function and DLS results in post-vaccination serum**

**Summary of correlations between outcomes of antibody function assays and aggregation**					
**Immunoglobulin activity assay**			**Correlation to v8 Size (nm)**		**n: Donors with correlated data**
**Viral strain/Epitope**	**Assay type**	**Isotype**	**Pearson R**	**Adj. P value**	
ConS gp140	Antigen binding	IgA	−0.538	0.014	20
NL4-3	Virus capture	IgG	−0.486	0.022	22
A244 gp120 gD-	Antigen binding	IgA	−0.461	0.041	20
gp120 C5 domain	Epitope mapping	IgG	0.591	0.056	11
A244	Virus capture	IgG	−0.441	0.067	18
TH023	Neutralization	Mixed	−0.261	0.171	29
MN gp120	Antigen binding	IgA	−0.308	0.186	20
gp120 C1 domain	Epitope mapping	IgG	0.373	0.259	11
MN	Virus capture	IgG	−0.205	0.372	21
MN	Virus capture	IgA	0.169	0.398	27
A244 gp120 gD+	Antigen binding	IgA	−0.153	0.519	20
MN	Neutralization	Mixed	−0.116	0.548	29
gp120 V3 domain	Epitope mapping	IgG	0.182	0.593	11
A244 gD+	ADCC	IgG	0.080	0.684	28
gp120 V2 domain	Epitope mapping	IgG	0.127	0.709	11
WITO	Virus capture	IgG	0.086	0.735	18
WITO	Virus capture	IgA	−0.077	0.742	21
NL4-3	Virus capture	IgA	−0.059	0.772	27
CM243	ADCC	IgG	−0.027	0.891	29
Multiple strains	Binding breadth	IgA	−0.022	0.911	29

Results for ADCC, neutralization and IgG Virion capture have been previously reported [27,29,32].

To summarize the results of the RV144 samples, serum IgA incubated with purified HIV-1_A244_ virions resulted in a statistically significant increase in particle size in 31.82% of vaccinees. Increased ability to affect virion size was paralleled by an increase in capture of infectious virions after vaccination however there was not direct correlation between the two. The moderate effect of aggregation using these samples is likely due to the low concentration of dimeric relative to monomeric IgA in the serum.

## Discussion

We have assessed for the first time antibody characteristics determining the potential to form aggregate complexes with HIV-1. Initial experiments with soluble trimeric Env and IgG mAbs demonstrate how the stoichiometry of aggregation is an important consideration: excess antigen or antibody reduces the potential for complexes to form, typical of a prozone effect. Most Env specific IgGs were able to form Env-aggregates to some degree, but efficiency and optimal concentration varied significantly. These data indicate that aggregate formation is likely determined by multiple binding modes. The same IgG mAbs were unable to aggregate virions. Thus while Env specific IgGs possess the intrinsic capacity to bridge between trimers, they are unable to stably bind trimers on opposing viral membranes.

The inability of IgG to aggregate HIV-1 most likely reflects steric restriction based on presentation of the trimer within the viral membrane precluding favorable mAb approach angles. The inability to aggregate virions is not related to monoclonality as polyclonal HIV-Ig was also unable to aggregate HIV. Aggregation requires monovalent antibody binding with an orientation where one of the two Fab arms is directed towards the solution and available for bridging. This is compounded by the low density of Env spikes on individual virions (7-15) and the distance between membrane-anchored Env complexes on opposing virions. An alternative scenario would be that of bivalent binding of individual HIV mAbs, either to the same trimer or between trimers on the same virion precluding availability of free Fab arms for aggregation, however affinity studies suggest this is not the case [33]. Indeed heteroligation of Env spikes has only been reported for chimeric bispecific antibodies [34]. Furthermore, the observation that aggregation could be induced by secondary addition of anti-IgG antibody suggests that neither spike density nor the distance between Env complexes on opposing virions is limiting (the binding of the primary Env mAb being directly proportional to spike density). These data demonstrate effective opsonisation of virus by IgG and add support to the hypothesis that the Fab approach angle is likely the dominant factor in determining virion aggregation. These data are in line with earlier studies implying the angle of approach between epitope and Ab is the principle determinant for the potential to aggregate poliovirus [35]. The shape of IgG likely restricts positioning of Fabs on the Env trimer, causing the second free Fab arm to be unavailable for interaction with a second trimer. In contrast to IgG, IgA_1_ has a longer hinge region that may have reduced steric restrictions, however class switched monomeric versions of 2F5 IgA_1_ was also unable to aggregate HIV despite having similar ability to bind whole virions as their IgG counterparts.

In contrast to monomeric immunoglobulins, both dimeric 2F5 IgA_1_ and b12 IgA_2_ were capable of inducing aggregation. Here, two monomers are connected end-to-end through their Fc regions by a flexible joining chain (J chain). As a result the Fabs of each monomer are diametrically opposed, with access to a broader array of orientations. Thus the Fabs of each monomer are able to have favorable and independent approach angles to trimers on different virions [36]. However the proportion of virions aggregated and the size of higher order complexes were influenced by antibody specificity.

2F5 dIgA_1_ was able to aggregate up to 50% of virions into aggregates of 6-8 or 24-30 virions. This suggests the low affinity reactivity of 2F5 against membrane lipids appeared insufficient to cause pan-virion aggregation [37]. The observation of two discrete orders of aggregation, rather than a dispersed size distribution, suggests these two populations may reflect differences in epitope exposure on discrete virion populations. The principal epitope of 2F5 mAb is within the MPER of gp41. This is thought to be occluded on functional trimers of primary virus and on HIV-1 BaL used here [38], but is transiently exposed on receptor/coreceptor binding [39]. However, the 2F5 epitope would be accessible on non-functional Env forms such as non-cleaved gp160 and those gp41 molecules undergoing spontaneous triggering, having shed their gp120. Whether the two aggregate populations represent different presentations of gp41 or levels of MPER exposure requires further investigation. An alternative explanation would be the difference in the distribution of Env spikes on immature and mature particles, being randomly distributed on the former, while tending to cluster into a single small domain with decreased inter-spike distances on the latter [40]. This is less likely given that this would similarly influence b12 dIgA_2_. Indeed b12 dIgA_2_ produced a single aggregate peak of 6-8 virions that comprised 35-40% of total virions. It is unclear at this stage whether the differences in formation of higher order aggregates between 2F5 IgA_1_ and b12 IgA_2_ reflect difference in antigen specificity or differences in hinge length and rigidity between IgA_1_ and IgA_2_. These preliminary data suggest that aggregation is dependent upon the distribution of epitope exposure across a virion population. Study of a wider panel of dIgA of varied specificity with matched IgA_1/2_ isotypes is now needed to understand the impact on formation of aggregate complexes.

Analysis of serum IgA samples from RV144 vaccinated subjects with high systemic IgA binding antibody demonstrated a small but highly statistically significant increase in complex size. Of the 22 measurements tested for correlation with aggregation, 3 measurements involving binding and virion capture were negatively correlated with virion aggregation (p < 0.05, adjusted for multiple comparisons; R values of -0.461 to -0.538). This weak correlation supports the finding that virion aggregation is a distinct immune measurement from binding and virion capture. The RV144 + gD gp120 immunogen containing an N terminal 11 amino acid deletion was associated with enhanced antigenicity for C1, V2, and V1/V2 conformational epitopes [41]. Thus, the observed negative correlation with binding to gD- A244 and ConS gp140 might be a surrogate for lower levels of IgA antibodies with the epitope specificity required for efficient aggregation. The negative correlation with IgG viral capture of NL4-3 is less clear. Interestingly, high levels of serum binding IgA specific for variable regions 1 and 2 (V1V2) of gp120 were correlated with reduced vaccine efficacy [42]. One proposed explanation is that monomeric Env-specific IgA blocked the binding of C1 specific ADCC mediating IgG [28]. We find no correlation between the DLS results and C1 binding. Our observation that specific IgA effectively opsonized A244 virions suggests an alternative but not mutually exclusive mechanism: IgA may diminish specific IgG binding and as a consequence reduce FcγR mediated phagocytosis and/or complement fixation. This raises critical questions as to the influence of IgA responses for HIV vaccine efficacy in general. While vaccinations in the RV144 trial also included HIV-1 strain MN, there was insufficient purified serum IgA available to assess it potential aggregation.

The majority of the IgA in the serum is monomeric and, based on data from these studies, would opsonize but not aggregate virions. Monomeric IgA is unable to trigger complement or engage FcγR antiviral activity, thus competition with IgG for binding might be detrimental. However the concentration of serum IgG is on average 4-fold higher than IgA. For specific monomeric IgA to have a significant impact it would require an affinity sufficient to outcompete the higher concentration of IgG. It would also be premature to exclude an antiviral role of the FcαR on IgA opsonized virions.

At mucosal surfaces IgA is expressed in its dimeric form with potential to induce virion aggregation and enabling immune exclusion. In this study we have examined the potential of two neutralizing antibodies to aggregate HIV-1. These initial studies indicate different forms of aggregation based on epitope specificity and isotype (IgA_1_/IgA_2_). Viral aggregation may augment the protective efficacy of neutralizing antibodies. This is supported by a recent study in rhesus macaques demonstrating that the dIgA_1_ isoform of the neutralizing MAb HGN194 afforded the best *in vivo* protection (relative to IgG1 and dIgA2) against rectal challenge [43]. The extent to which non-neutralizing antibodies might mediate similar protective effects is likely dependent upon their epitope expression on infectious virions.

## Conclusions

In summary, to the best of our knowledge, this study demonstrates for the first time the capacity of Env-specific antibodies to cross-link HIV-1 virions into aggregates. The ability to form aggregated immune complexes was determined by the valency of the antibody, effectively induced by polymeric but not monomeric isoforms. Both the degree and higher order structure of aggregate formation was seen to vary based the epitope specificity of the dIgA. Indeed, the two neutralizing dIgAs aggregated a subset of virus reflective of the population of virions containing forms of Env exposing their specific epitope. Further studies are now needed to determine the impact of non-neutralizing dIgA on aggregation of infectious virions to determine whether this function is synonymous or independent of neutralization. Furthermore, mucosal sampling within clinical trials, and specifically follow on studies from the RV144 protocol, will be important to understand the mechanism by which IgA responses may enhance or subvert protective responses.

## Methods

### Materials

Antibodies were obtained from Polymun Scientific (Vienna, Austria) unless otherwise specified. 2F5 IgG, IgA, and IgM were purchased from Polymun (Vienna, Austria) and shared through the CHAVI consortium. Antibodies b12 IgG, monomeric b12 IgA (mIgA) and dimeric IgA (dIgA) were donated by Dennis Burton. CN54 gp140 was chosen for analysis based on availability of clinical grade material and was also obtained from Polymun Scientific. The following reagents were obtained through the AIDS Research and Reference Reagent Program, Division of AIDS, NIAID, NIH: HIV-1 strains BaL and RF are donated by Dr. Suzanne Gartner, Dr. Mikulas Popovic and Dr. Robert Gallo; T cell line PM1 was donated by Dr. Marvin Reitz; HIV-Immunoglobulin (HIV-Ig) was obtained from NABI and National Heart Lung and Blood Institute from Dr. Luiz Barbosa. Serum IgA samples were isolated from RV144 study participant samples and shared through the CHAVI consortium. Human isotype controls were purchased from Sigma-Aldrich.

### Viral culture and purification

Chronically infected PM-1 cells were established following infection with HIV-1_BaL_ or HIV-1_RF_ as previously described [44]. Strain HIV-1_A244_ was grown in PBMCs pooled from 10 donors and collected at peak viral antigen production. Viral production was quantified by viral capsid (p24 antigen) release, as measured by ELISA (NCI Frederick, MD) according to the manufacturer’s protocol. Supernatants were harvested to produce a viral stock when p24 levels were greater than 250 ngml^−1^. Virions were inactivated by incubation with aldrithol-2 (AT-2) for 1 h at 37°C [45].

To purify virions and microvesicles from other lipid vesicles, a protocol similar to those previously described was used [24]. Briefly, cell supernatant stocks were layered on top of a 17-25% sucrose solution and spun at 100,000 g in a SW55Ti rotor until pelleted. Supernatants were aspirated, and pellets were resuspended in PBS supplemented with 1% bovine serum albumin (BSA) and 5 mM EDTA (Sigma-Aldrich). CD45 conjugated magnetic beads (Miltenyi Biotec) were added at 10 μlml^−1^ relative to initial stock. This preparation of lipidic vesicles was incubated at 4°C with gentile mixing for a 4 to 6 hours before depletion according to the manufacturer’s instructions. Immune depleted virions were pelleted at 150,000 x g on 25% sucrose cushions for 60 minutes and resuspended in PBS for analysis. Purified HIV-1_BaL_ and HIV-1_RF_ preparations had a Z-average diameter of 150.2nM with a polydispersity index of 0.150, in agreement with previous descriptions of viral preparations [21,24].

### Binding kinetics

Determinations of the affinity and relative avidity of HIV-1 virions for monoclonal antibodies 2F5 and b12 were assessed on a RapID4 acoustic biosensor (TTP Labtech). Antibody of interest and an inert control of each isotype were bound on the flow cell surface using a carbodiimide (EDC) and *N*-hydroxysuccinimide (NHS) linkage. All procedures were performed at a flow rate of 25μlmin^−1^. Antibody to be coupled was diluted to a concentration of 50 μgml^−1^ in 100 mM sodium acetate at pH 4.5. Capping was performed with 0.5 M ethanolamine, pH 8.0. Sensor cassettes were then conditioned with 100 mM glycine, pH 2.5 with 0.025% Tween 20.

Virions were allowed to flow over the Ab coated surface at a range of concentrations from 10 ngml^−1^ to 1.0 μgml^−1^ p24 in PBS and for 3 minutes, followed by dissociation. Surfaces were regenerated by washing with 100 mM glycine buffer at a pH of 2.5 with 0.1% Tween 20. Experiments were performed in triplicate. Affinities for all virus-antibody binding systems were calculated according to the Langmuir kinetic model.

### Dynamic light scattering size measurements

#### Assay development

Viral aggregation has not previously been studied for HIV-1 or to our knowledge, any other enveloped viruses aside from analysis by electron microscopy. To make measurements of aggregate formation for many antibodies at a range of concentrations required developing a novel methodology. Size determination was carried out using a Malvern ZetaSizer ZS Nano (Malvern Instruments, England), using ZetaSizer software v7.01 in dual angle (173° backscatter and 7° forward scatter) light scattering mode. Samples were maintained at 37°C before and during measurements. Preparations were considered to be composed primarily of protein, so that the refractive indexes of samples could be held constant. The light scattering technique uses a time-correlation function to determine the size of suspended particles diffusing due to Brownian motion. Samples that self correlate for longer time scales are larger than ones where the time-correlation function decreases rapidly.

The slope of the time-correlation function determines the polydispersity of a sample. Materials that are homogeneous will have a more steeply sloped time-correlation function (and lower polydispersity index, (PDI)) than heterogeneous ones. This value ranges from 0 for a perfectly homogeneous sample, to 1 for a random distribution of sizes. Differences in the PDI can be used to assess changes in heterogeneity between similarly prepared samples. The value of the PDI is not indicative of any absolute property, but measures the degree of “sameness” within a sample.

Assay development proceeded from initial measurements that were based on the concentration of virions used to determine that a single population of particles was present in the purified viral preparations. Purified virions were used at a concentration of 25 μgml^−1^ p24 protein. Aggregate formation was stable from 1 hour up to 6 hours at 37°C, although lower temperatures did not abrogate aggregate formation with all antibodies tested. Extended time frames resulted in variability in readings, attributable to decreased viral integrity over time.

While virions were incubated a range of antibody concentrations, results reported are exclusively from 10 μgml^−1^, as this was most favorable for complex formation. When a secondary anti-human or anti-murine IgG antibody (Sigma-Aldrich, St. Louis, USA) was used, after incubation of virus-antibody preparations, free antibody was removed by passing the sample through a 250kD spin column (Promega, Madison, USA). Secondary antibody was added at a concentration of 5 μgml^−1^ and samples were incubated for 30 minutes at 37°C. Sizing measurements were made in the same manner as for virus-antibody preparations.

#### SEC-DLS determination of viral aggregation

DLS measurements alone are very precise for measurement of the size of a monodisperse suspension. When multiple species are present, such as in the case of viral aggregates in the presence of non-aggregated virions, DLS accuracy decreases. Separation of the components of a complex mixture by size exclusion chromatography (SEC), followed by DLS measurements of each fraction allows accurate characterization of each species present. Antibody and purified virus complexes were prepared according to the same antibody-virus ratios as for bulk DLS measurements. Separation was done on a TOSOH G4000SW SEC using a Gilson HPLC system with model 305 pumps with a manual injection loop. A mobile phase of PBS with 0.05 mM EDTA was used at a flow rate of 500 μlmin^−1^. DLS measurements were acquired continuously and stored every 3 seconds with ZetaSizer software v7.01.

### Binding antibody assessment

Binding antibody multiplex assays for IgG and IgA and purification of plasma IgA were performed as previously described [28,46]. Plasma IgA was purified by peptide M purification as previously described [28]. Confirmatory IgA and IgG binding assays demonstrated Env specific IgA binding and depletion of IgG below detectable levels.

### Microplate infectious virion capture assay

Infectious virion capture assay was performed as previously described [29]. Briefly, Microplates (NUNC) were coated overnight at 4°C with Goat Anti-Human Serum IgA (Jackson ImmunoResearch Laboratories) at a concentration of 2 μgml^**−1**^ diluted in PBS. After coating and washing, coated plates were blocked for 2 hr with PBS supplemented with 5% Goat serum, 5% milk, 0.05% Tween. The indicated concentration of antibodies was mixed with the viral stock containing 1.5 x 10^7^ viral RNA (measured by RT-PCR) and then centrifuged 90 min at 2,000 rpm. Then the mixture was centrifuged at 21,000 x g for 1 hour at 4°C to remove the virus-free antibody, the pellet was resuspended in the same volume of PBS. 50 μl of the immune complex mixture was added to each coated well in triplicate wells for 90 min. The wells were washed 4 times and the susceptible target cells (M7-Luc indicator cells or A3R5 cells (for virus T/F WITO)) in 100 μl were added. HIV-1 infection was assessed on day 7 after infection for M7-Luc and on day 6 for A3R5 cells by luciferase assays. For M7-Luc firefly luciferase assay, 100 μl supernatant was removed, and 100 μl Britelite (Perkin Elmer) was added to each well. After 2-min incubation, the 150 μl lysate was used to measure HIV-1 infection as expressed as relative luciferase units (RLUs). For A3R5 Renilla luciferase assay, luciferase expression of infected cells was measured with the Renilla luciferase assay kit (Promega) and read on a Berthold E & G luminometer using MicroWin software.

### Statistical analysis

The statistical significance of results was determined by nonparametric analysis employing Wilcoxon’s matched pairs, Pearson’s correlation, or 1-way ANOVA with the appropriate subtest as specified using GraphPad Prism 5.0 software (Graphpad Software Inc., La Jolla, CA). Multiple Pearson regression analysis of antibody functions was performed in R using and the statistical significance of the correlations was adjusted by the Bonferonni correction.

## Additional files

Additional file 1: Figure S1. Addition of a secondary anti-Fc antibody to IgG opsonized virions induces aggregation. HIV incubated with human (A) 1F7, (B) HIV-Ig, or (C) anti-HLA-DR antibody followed by removal of free antibody, and addition of a secondary anti-Fc. Complex size is measured in triplicate by DLS. Data shown represent the mean of three experiments. Additional file 2: Figure S2. HIV-1 (A) BaL and (B) RF binds specifically to 2F5 and b12 after isotype switching. Binding between b12 and 2F5 antibodies of IgG, dimeric IgA and pentameric IgM isotypes to virions was measured on an Akubio RapID4 analyzer, with each antibody covalently attached to a sensor cassette. Purified HIV-1_BaL_ virions were allowed to bind for 3 minutes, followed by 10 minutes of dissociation. Affinities were calculated according to a Langmuir binding model. Measurements were made in triplicate and data shown is representative from one experiment. Additional file 3: Figure S3. Size-exclusion chromatography and dynamic light scattering in tandem enable characterization of the viral complexes formed with 2F5 IgG. Virus incubated with 2F5 IgG was separated over a size exclusion column and followed by absorbance measured by (A) infrared, (B) ultraviolet, and (C) DLS detection. (D) Size was determined from the peak eluted from the SEC system. One peak was detected with the IgG preparation, eluted at 23.5 minutes and measuring 176 nm. Experiments were performed in triplicate. Results shown are from one representative experiment. Additional file 4: Figure S4. Size-exclusion chromatography and dynamic light scattering in tandem enable characterization of viral complexes formed with b12 IgG. Virus incubated with b12 IgG was separated over a size exclusion column and followed by absorbance measured by with (A) infrared, (B) ultraviolet and (C) DLS detection. (D) Size was determined from the one peak eluted from the SEC system. One peak was detected with the IgG preparation, eluted at 23 minutes and measuring 176 nm. Experiments were performed in triplicate. Results shown are from one representative experiment. Additional file 5: Figure S5. Western blots for IgA of purified IgA from 8 patients in the RV144 trial. IgA is separated on an 8% Native-PAGE gel and transferred to a PVDF membrane. Plot is probed with goat anti-human-IgA. Monomeric and dimeric forms of IgA are seen in both the pre and post-immunization samples. Additional file 6: Figure S6. Size distribution of purified HIV-1_A244_ and IgA preparations from 8 patients. Size distribution of purified virions and IgA isolated from 8 patient sera was measured using dynamic light scattering. (A) Three preparations of purified virions were measured by DLS and had similar size distributions, with a Z-average diameter of 216.6 nm. (B) Pre-immunization serum from 8 patients had the same size distribution, with particles ranging from 6 to 12 nm in diameter. Results shown are the average of three experiments, each measured in triplicate.

## Abbreviations

Ab: Antibody
ADCC: Antibody Dependent Cellular Cytotoxicity
bnAb: Broadly-neutralizing Antibody
CD4bs: CD4 binding site
dIgA: Dimeric IgA
DLS: Dynamic Light Scattering
Env: HIV-1 Envelope Glycoprotein
HIV-1: Human Immunodeficiency Virus 1
IgA: Immunoglobulin A
IgG: Immunoglobulin G
IgM: Immunoglobulin M
mAb: Monoclonal Antibody
MPER: Membrane Proximal External Region
nAb: Neutralizing Antibody
nnAb: Non-neutralizing Antibody
PDI: Polydispersity Index
pIgR: Polymeric Immunoglobulin Receptor
scIgA: Secretory IgA
SEC: Size Exclusion Chromatography
