# Supplementary figures and images for: Aggregate complexes of HIV-1 induced by multimeric antibodies
Source: Retrovirology. 2014 Oct 2;11:78. doi: 10.1186/s12977-014-0078-8 (PMC4193994; doi:10.1186/s12977-014-0078-8)

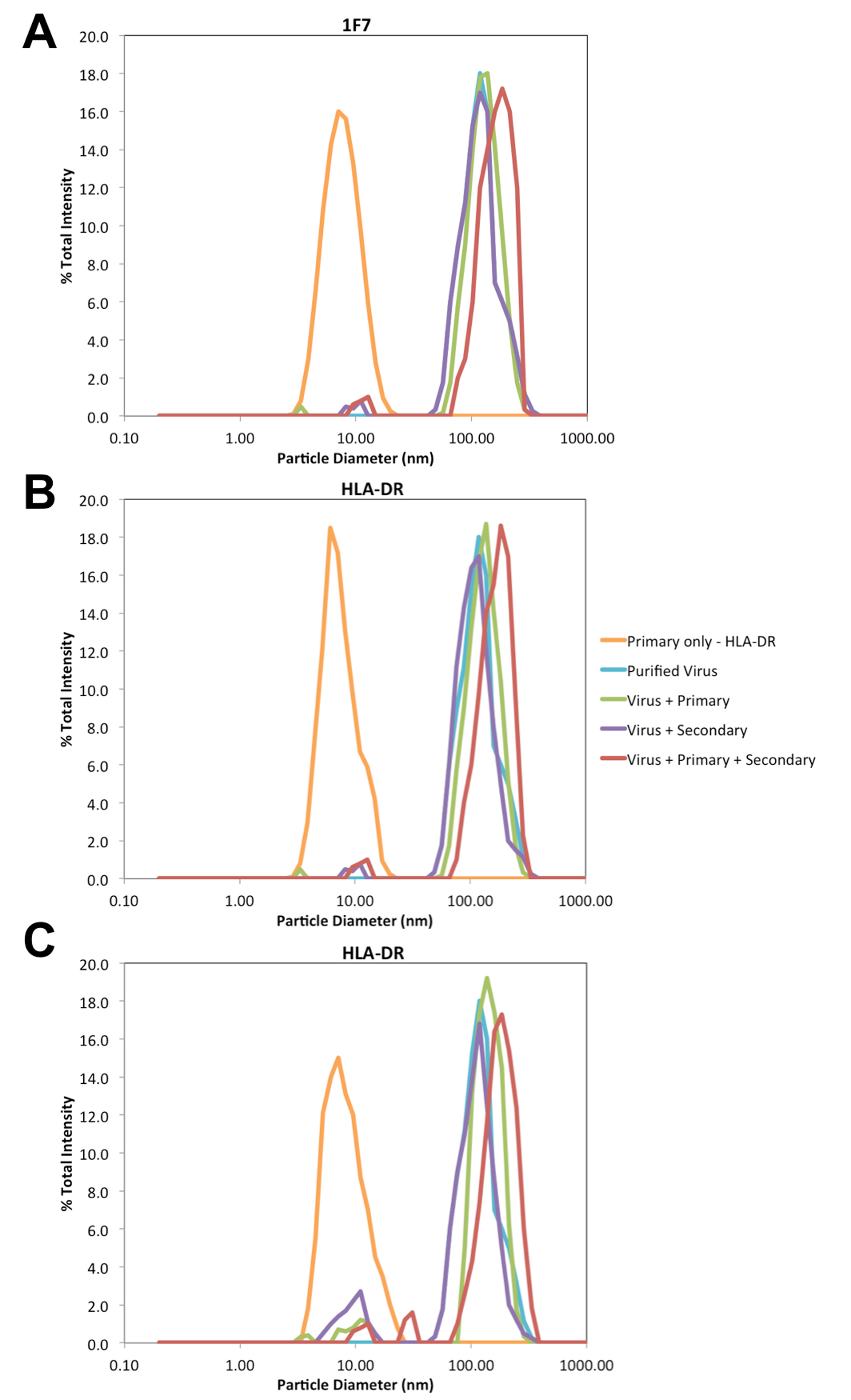

Supplement: Additional file 1: Figure S1. — Addition of a secondary anti-Fc antibody to IgG opsonized virions induces aggregation. HIV incubated with human (A) 1F7, (B) HIV-Ig, or (C) anti-HLA-DR antibody followed by removal of free antibody, and addition of a secondary anti-Fc. Complex size is measured in triplicate by DLS. Data shown represent the mean of three experiments. [file 12977_2014_78_MOESM1_ESM.tiff]

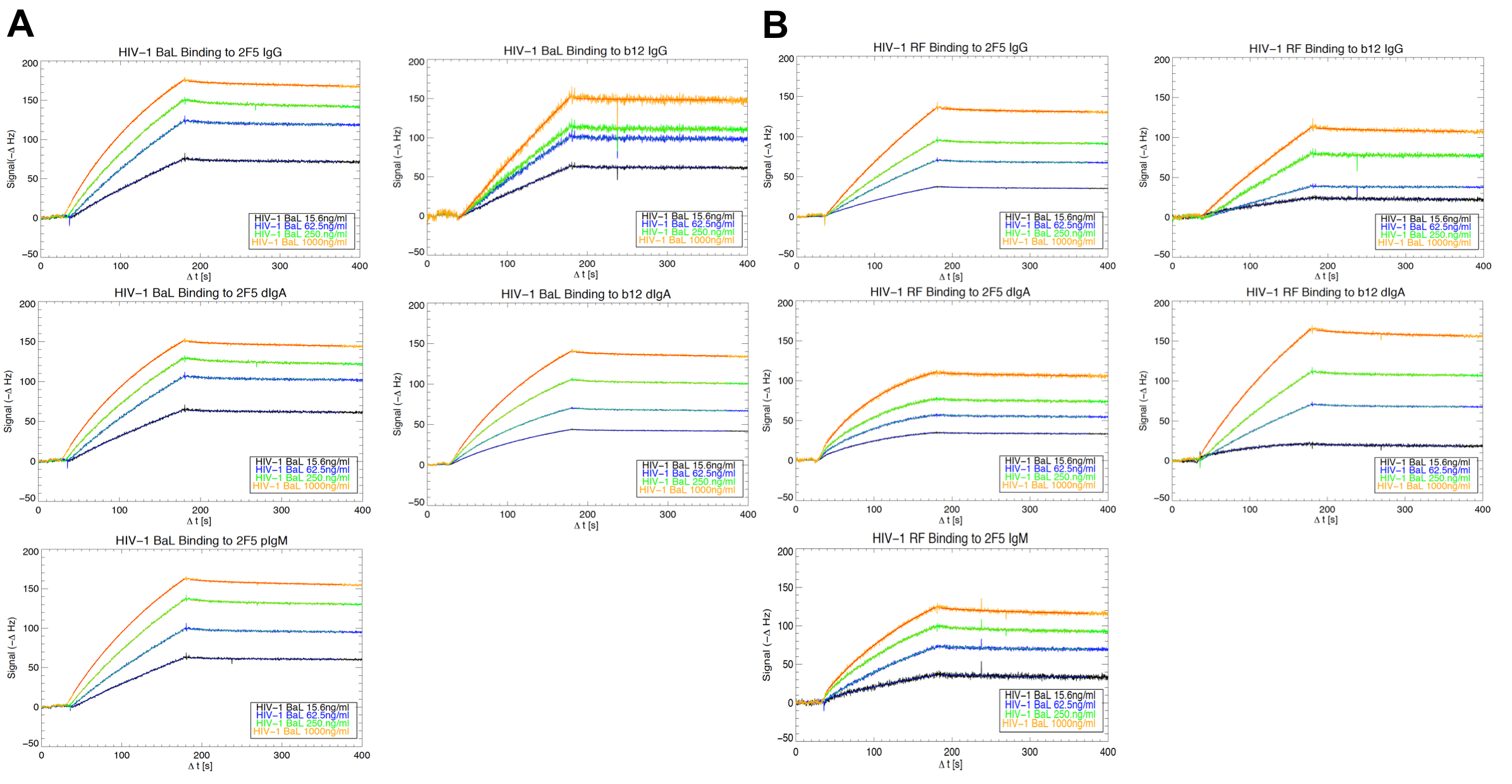

Supplement: Additional file 2: Figure S2. — HIV-1 (A) BaL and (B) RF binds specifically to 2F5 and b12 after isotype switching. Binding between b12 and 2F5 antibodies of IgG, dimeric IgA and pentameric IgM isotypes to virions was measured on an Akubio RapID4 analyzer, with each antibody covalently attached to a sensor cassette. Purified HIV-1BaL virions were allowed to bind for 3 minutes, followed by 10 minutes of dissociation. Affinities were calculated according to a Langmuir binding model. Measurements were made in triplicate and data shown is representative from one experiment. [file 12977_2014_78_MOESM2_ESM.tiff]

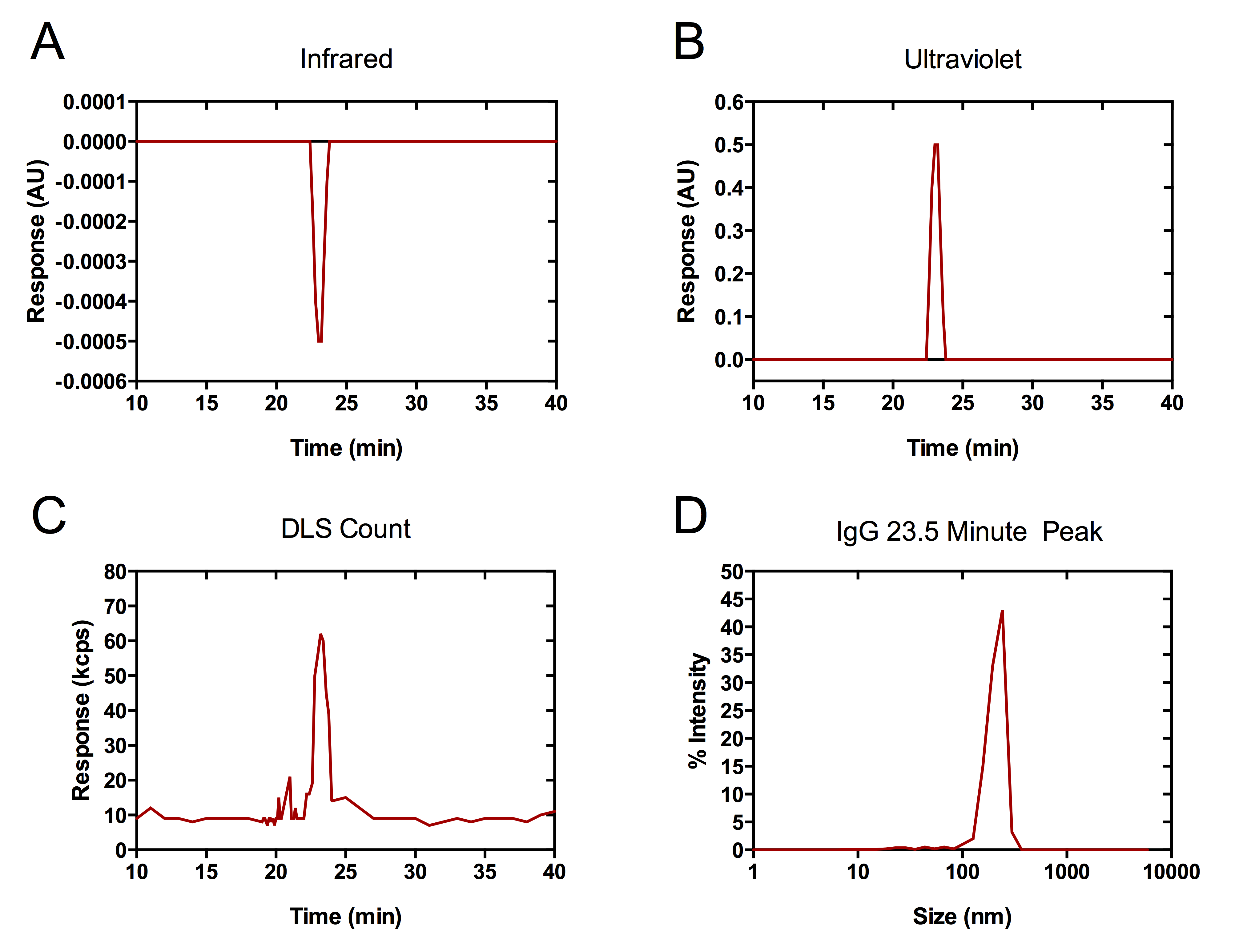

Supplement: Additional file 3: Figure S3. — Size-exclusion chromatography and dynamic light scattering in tandem enable characterization of the viral complexes formed with 2F5 IgG. Virus incubated with 2F5 IgG was separated over a size exclusion column and followed by absorbance measured by (A) infrared, (B) ultraviolet, and (C) DLS detection. (D) Size was determined from the peak eluted from the SEC system. One peak was detected with the IgG preparation, eluted at 23.5 minutes and measuring 176 nm. Experiments were performed in triplicate. Results shown are from one representative experiment. [file 12977_2014_78_MOESM3_ESM.tiff]

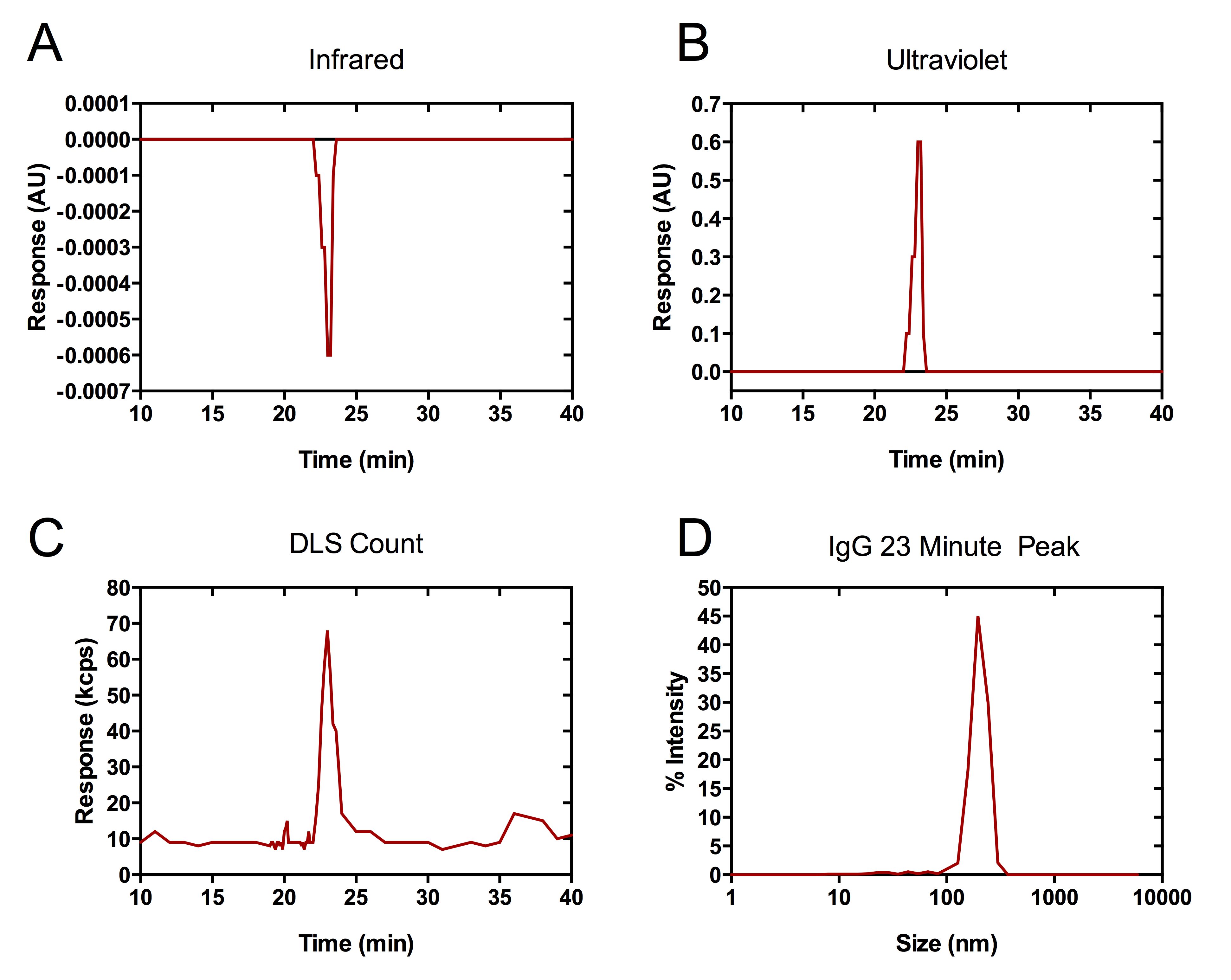

Supplement: Additional file 4: Figure S4. — Size-exclusion chromatography and dynamic light scattering in tandem enable characterization of viral complexes formed with b12 IgG. Virus incubated with b12 IgG was separated over a size exclusion column and followed by absorbance measured by with (A) infrared, (B) ultraviolet and (C) DLS detection. (D) Size was determined from the one peak eluted from the SEC system. One peak was detected with the IgG preparation, eluted at 23 minutes and measuring 176 nm. Experiments were performed in triplicate. Results shown are from one representative experiment. [file 12977_2014_78_MOESM4_ESM.tiff]

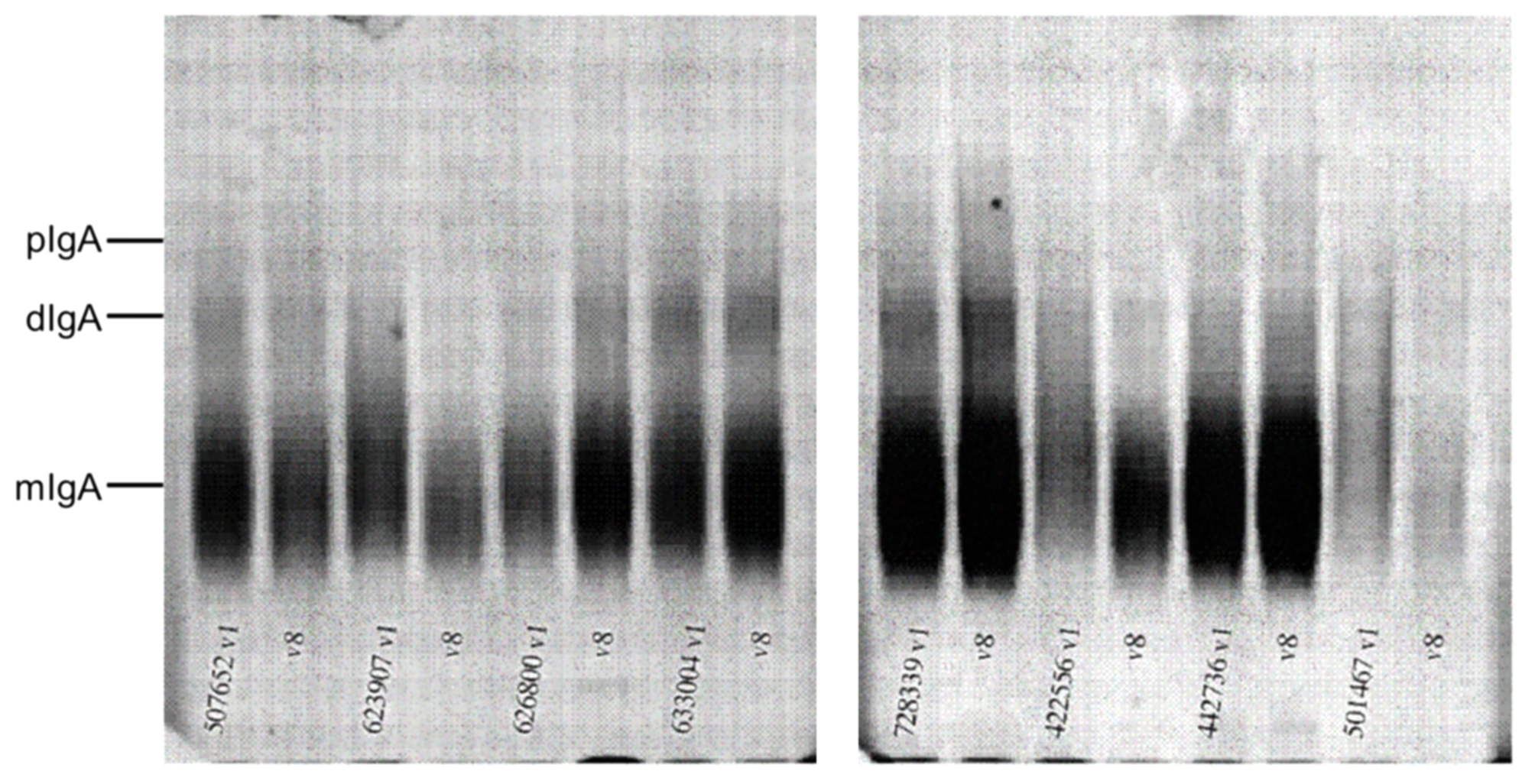

Supplement: Additional file 5: Figure S5. — Western blots for IgA of purified IgA from 8 patients in the RV144 trial. IgA is separated on an 8% Native-PAGE gel and transferred to a PVDF membrane. Plot is probed with goat anti-human-IgA. Monomeric and dimeric forms of IgA are seen in both the pre and post-immunization samples. [file 12977_2014_78_MOESM5_ESM.tiff]

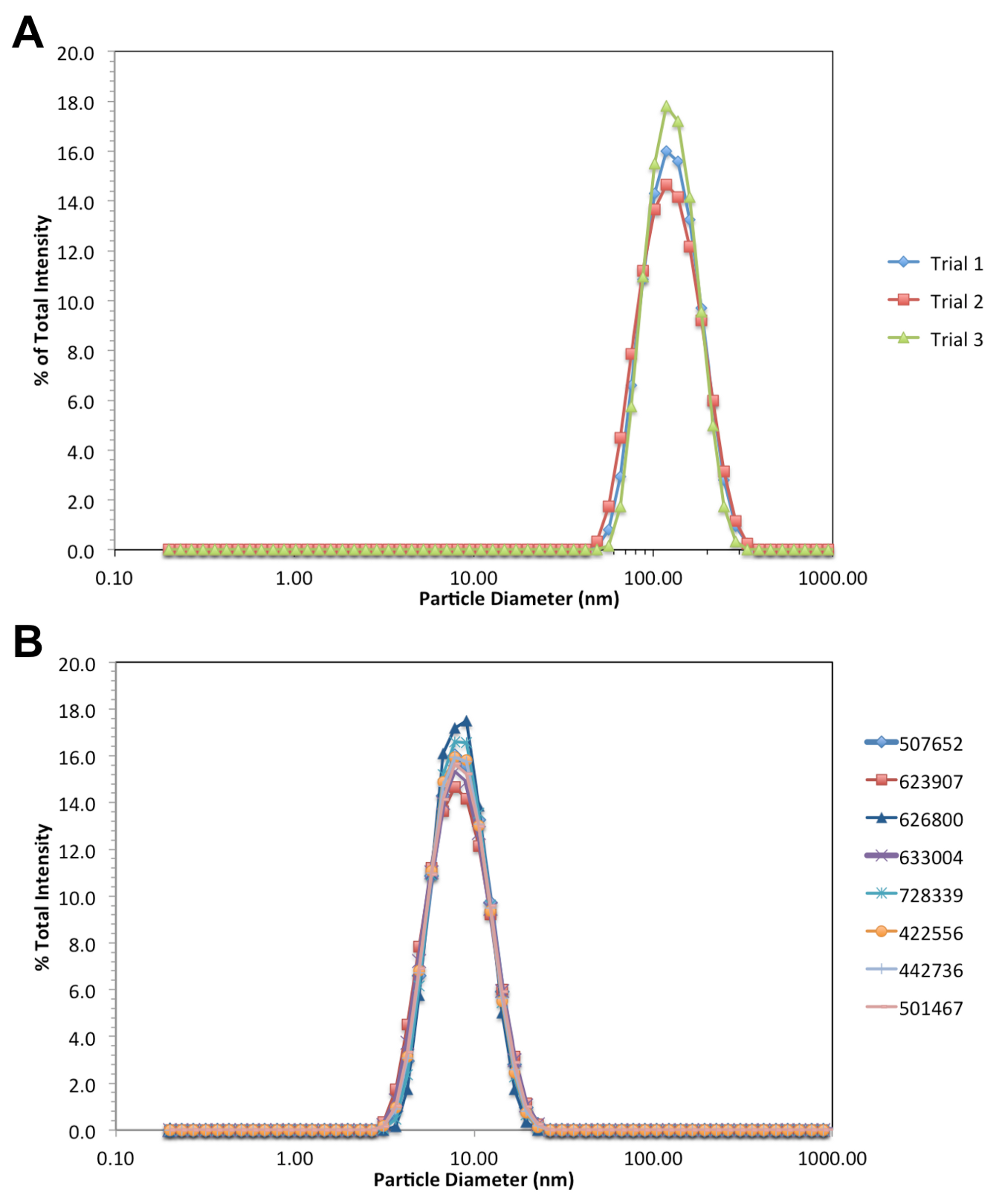

Supplement: Additional file 6: Figure S6. — Size distribution of purified HIV-1A244 and IgA preparations from 8 patients. Size distribution of purified virions and IgA isolated from 8 patient sera was measured using dynamic light scattering. (A) Three preparations of purified virions were measured by DLS and had similar size distributions, with a Z-average diameter of 216.6 nm. (B) Pre-immunization serum from 8 patients had the same size distribution, with particles ranging from 6 to 12 nm in diameter. Results shown are the average of three experiments, each measured in triplicate. [file 12977_2014_78_MOESM6_ESM.tiff]
